# Sex classification using the human sacrum: Geometric morphometrics versus conventional approaches

**DOI:** 10.1371/journal.pone.0264770

**Published:** 2022-04-06

**Authors:** Viktoria A. Krenn, Nicole M. Webb, Cinzia Fornai, Martin Haeusler

**Affiliations:** 1 Institute of Evolutionary Medicine, University of Zurich, Zurich, Switzerland; 2 Department of Evolutionary Anthropology, University of Vienna, Vienna, Austria; 3 Human Evolution and Archaeological Sciences (HEAS), University of Vienna, Vienna, Austria; 4 Senckenberg Society for Nature Research, Leibniz Institution for Biodiversity and Earth System Research, Frankfurt, Germany; 5 Institute of Archaeological Sciences, Senckenberg Centre for Human Evolution and Palaeoenvironment, Eberhard Karls University of Tübingen, Tübingen, Germany; 6 Vienna School of Interdisciplinary Dentistry, Klosterneuburg, Austria; Liverpool John Moores University, UNITED KINGDOM

## Abstract

The human pelvis shows marked sexual dimorphism that stems from the conflicting selective pressures of bipedal locomotion and parturition. The sacrum is thought to reflect this dimorphism as it makes up a significant portion of the pelvic girdle. However, reported sexual classification accuracies vary considerably depending on the method and reference sample (54%-98%). We aim to explore this inconsistency by quantifying sexual dimorphism and sex classification accuracies in a geographically heterogeneous sample by comparing 3D geometric morphometrics with the more commonly employed linear metric and qualitative assessments. Our sample included 164 modern humans from Africa, Europe, Asia, and America. The geometric morphometric analysis was based on 44 landmarks and 56 semilandmarks. Linear dimensions included sacral width, corpus depth and width, and the corresponding indices. The qualitative inspection relied on traditional macroscopic features such as proportions between the corpus of the first sacral vertebrae and the alae, and sagittal and coronal curvature of the sacrum. Classification accuracy was determined using linear discriminant function analysis for the entire sample and for the largest subsamples (i.e., Europeans and Africans). Male and female sacral shapes extensively overlapped in the geometric morphometric investigation, leading to a classification accuracy of 72%. Anteroposterior corpus depth was the most powerful discriminating linear parameter (83%), followed by the corpus-area index (78%). Qualitative inspection yielded lower accuracies (64–76%). Classification accuracy was higher for the Central European subsample and diminished with increasing geographical heterogeneity of the subgroups. Although the sacrum forms an integral part of the birth canal, our results suggest that its sex-related variation is surprisingly low. Morphological variation thus seems to be driven also by other factors, including body size, and sacrum shape is therefore likely under stronger biomechanical rather than obstetric selection.

## Introduction

The sacrum plays a fundamental biomechanical role within the body and is often considered the "inverted keystone" of the pelvis in analogy with the keystone at the apex of a masonry arch [see 1–3]. In fact, it comparably provides effective weight transmission, in this case to the lower limbs, while simultaneously preventing the collapse of the pelvic ring via the strong interosseous and posterior sacroiliac ligaments [see 1]. In addition, the sacrum forms part of the birth canal and thereby contributes to pelvic sexual dimorphism [e.g., 4, 5–18]. Reported sexual classification accuracies for the human sacrum range from 54% to 98% depending on the study (see Table 1 for an overview of the literature) and thus are generally lower than the sexual classification accuracies obtained for the hip bones (between 90% and 100%) [19–22]. This is unexpected given that biomechanical and obstetrical constraints apply to both the sacrum and the hip bones as each of these elements collectively contributes to the transmission of body weight and the shape of the birth canal. Yet, sacrum morphology seems to vary in ways that remain poorly understood. An evaluation of the published evidence on the discriminative power of sacrum shape and form is difficult due to the heterogeneity of the methods used in the literature and the different geographical origins of the samples employed.

**Table 1:** Previously published classification accuracies (%) for sex determination based on the modern human sacrum.

Publication	Studied anatomical region	Method (Number of variables considered in the discriminant function)	Sample	Classification accuracy		
				F (n)	M (n)	Mean (n)
Anastasiou and Chamberlain [23]	Auricular surface	Centroid size based on 2D GM	British (archaeological, sex estimated)	75.9 (29)	82.9 (35)	79.4 (64)
		2D GM (shape variables plus centroid size)		79.3 (29)	88.6 (35)	84.0 (64)
Benazzi, Maestri [24]	Sacral base	Linear metrics (4 variables)	Italian (early 20th century)	87.5^§^ (56)	85.5^§^ (55)	86.5^§^ (111)
Etli, Asirdizer [25]	Complete sacrum	Linear metrics (21 variables)	Turkish (clinical)	82.1^§^ (240)	82.9^§^ (240)	82.5^§^ (480)
		Linear metrics (neural network)		87.2^§^ (240)	83.6^§^ (240)	85.4^§^ (480)
Flander [8]	Complete sacrum	Linear metrics (6 variables)	European Americans and African Americans ^‡^	88.0 (50)	80.0 (50)	84.0 (100)
				88.0 (50)	94.0 (50)	91.0 (100)
Gaya-Sancho, Aguilera [10]	Complete sacrum	Linear metrics (2 variables)	Spanish	95.6 (71)	67.2 (99)	81.4 (170)
Hegazy [26]	First sacral vertebra	Linear metrics (4 variables)	Arab (clinical)	92.5 (50)	97.5 (50)	95.0 (100)
Kimura [11]	Base-wing-index	Linear metrics (2 variables)	Japanese, European Americans and African Americans ^†^	N.A.	N.A.	75.3 (103)
				N.A.	N.A.	80.4 (100)
				N.A.	N.A.	82.7 (98)
Plochocki [27]	Anterior sacral curvature	Linear metrics (9 variables)	European Americans and African Americans ^‡^	70.2^§^ (59)	69.8^§^ (66)	70.0^§^ (125)
Rogers and Saunders [28]	Complete sacrum	Discrete traits (sacrum shape)	Canadian (19^th^ century)	N.A.	N.A.	94.1 (49)
Rusk and Ousley [15]	Complete sacrum	3D GM	European Americans and African Americans ^‡^	N.A.	N.A.	98.0^§^ (99)
				N.A.	N.A.	93.1^§^ (102)
Steyn and Isaac (2008)	Complete sacrum	Linear metrics (3 variables)	Greek	54.0 (78)	64.3 (72)	59.2 (150)
Strádalová [16]	Complete sacrum	Linear metrics (11 variables)	Czecho-Slovakian	N.A.	N.A.	85–89 (128)
Torimitsu, Makino [29]	Complete sacrum	Linear metrics (4 variables)	Japanese (clinical)	85.2^§^ (115)	81.7^§^ (115)	83.5^§^ (230)
Zech, Hatch [30]	Sacral base	Linear metrics (2 variables)	Swiss	83.0^§^ (38)	76.0^§^ (37)	79.5^§^ (75)
Zhan, Fan [18]	Complete sacrum	Linear metrics (4 variables)	Chinese (clinical)	86.9^§^ (160)	83.2^§^ (190)	85.0^§^ (350)

† Terry collection,

‡ Hamann-Todd collection,

§ cross-validated results.

Abbreviations: F, females; M, males; GM, geometric morphometrics; N.A., not available.

In general, three approaches have traditionally been used to distinguish the female from the male sacrum, including 1) qualitative inspection, 2) linear measurements, and 3) advanced quantitative methods, i.e., 2D and 3D geometric morphometrics (GM) and machine learning approaches. Qualitative investigation methods and visual assessment of the sacrum date back to the 19^th^ century [e.g., 31] and are mostly focused on the general shape of the sacrum, including the sagittal and coronal curvatures, the relative corpus-to-alae proportions, and the shape of the auricular surface (all anatomical terms used in this work are according to the international Terminologia Anatomica [32]). This approach is still commonly used for sex estimation of human remains and provides classification accuracy rates between 75% [33] and 94% [28]. Yet, the classification accuracy of linear dimensions varies considerably, ranging from 61% [34] to 95% [26], depending on the specific variable, or the combination of variables, used. The absolute corpus width is the most powerful single discriminator reported to date, followed by the corpus-to-ala ratio [e.g., 5, 7, 8, 16, 33, 35, 36]. Recently, more sophisticated approaches, such as 3D geometric morphometrics [15, 33] and multilayer perceptron neural networks [25], have yielded promising sexual classification accuracies between 86% and 98%. This seems to suggest that the accuracy of sex determination increases if the shape of the entire sacrum is considered. However, in an earlier study [33], we found that in Central Europeans even advanced shape analyses based on a large set of landmarks and semilandmarks on the entire sacrum did not yield better results than corpus width alone.

Therefore, sex classification accuracy might vastly differ based on the samples analysed. For example, European Americans and African Americans of the Hamann-Todd and Terry collections yielded different accuracy percentages [8, 11, 13, 15]. According to these studies, European Americans possess a deeper sagittal sacral curvature than African Americans, indicating that geographic origin significantly influences patterns of variation and must be considered in the analysis of sexual dimorphism in the pelvis. However, many populations remain scarcely represented in the literature on sacrum variation [but see 18, 29, 37]. Understanding the global pattern of sexual dimorphism is also essential for assessing specimens of unknown provenience or for supporting comparative studies on fossil sacra [e.g., 38].

Therefore, the current study addresses important methodological concerns relevant for accurate sex estimation based on the sacrum. Specifically, our aim is threefold: 1) to quantify the amount of sexual dimorphism in the modern human sacrum in a geographically heterogeneous sample; 2) to identify population-specific patterns of sexual dimorphism; and 3) to compare the classification accuracies of three different methods for sex estimation, namely, 3D geometric morphometrics, conventional linear metrics, and qualitative assessment.

## Materials and methods

### Study sample and data acquisition

Our sample consisted of 164 recent modern humans grouped into five broad geographical categories: 110 Central Europeans (including 58 specimens from the Weisbach collection, comprised mostly of military personnel of the Austro-Hungarian empire collected by Augustin Weisbach in the late nineteenth century and chosen for its excellent preservation and absence of skeletal pathologies [see 33]), 16 West Africans, 9 South-East Asians, 6 Native Americans from Tierra del Fuego, and 23 small-bodied individuals including 12 Khoesan and 11 Pygmies (see Table 2 and details in S1 Table). Sex was known from records for all but nine individuals that were included in the sample but not considered in the sex classification accuracy analyses.

**Table 2:** Sample composition by origin and by analysis.

Origin	F	M	U	Totals
European	51	52	7	110
West African	5	11		16
Khoesan & Pygmies	9	12	2	23
South-East Asian	2	7		9
Native American	4	2		6
**Totals**	71	84	9	164
Analysis				
Geometric morphometrics	46	55	6	107
Linear metrics	67	78		145
Qualitative investigation	70	84		154

Abbreviations: F, female; M, male; U, unknown.

The samples consisted of digital models generated both from medical CT data and 3D surface scans of skeletal collections. Seven sacrum models within the European sample were constructed from CT images (slice thickness < 1.4 mm) using Amira (www.fei.com). All necessary permits were obtained for the described study, which complied with all relevant regulations. Specifically, the digital patient data were provided by the General Hospital Vienna, Austria, in collaboration with Helmut Prosch (Department of Biomedical Imaging and Image-guided Therapy, Medical University of Vienna, Austria) after clearance by the Ethics Commission (votum 1196/2017) and subsequent full anonymization by the Data Clearing House of the Medical University of Vienna, Austria.

No ethical approval was required for the skeletal sample, which was handled based on the code of ethics of the Institute of Evolutionary Medicine, University of Zurich (see https://www.iem.uzh.ch/en/institute/iemcodeofethics.html). We used a Polymetric PT-M4c 3D surface scanner (www.polymetric.de) and the QTSculptor software to acquire high-resolution 3D polygon meshes of the sacra from the osteological specimens [39]. The Khoesan specimens were scanned at the Department of Evolutionary Anthropology, University of Vienna. These specific collections have since been closed for thorough provenance research in the context of colonial injustice. The Department of Evolutionary Anthropology, University of Vienna, declares that it is fully aware of the highly problematic acquisition circumstances regarding indigenous remains procured by the Austrian anthropologist Rudolf Pöch (1870–1921) during his expeditions to Oceania and South Africa between 1904 and 1909. Image data collected before the formal block of the collection are, however, open for study and their inclusion does not negate the ethical concerns associated with their acquisition or imply the authors in any way condone possible former injustices.

Shape variables derived from both CT-based image data and high-resolution 3D surface models have been shown to be of similar quality so that they can readily be combined in a single analysis [40–43]. The lower resolution of the clinical data sets was sufficient for inclusion in our study as the analyses did not require the assessment of small, discrete traits such as sulci or osteophytes.

As detailed in Table 2, some individuals had to be excluded from the geometric morphometric analyses for the presence of vertebral segmentation anomalies, preventing the sampling of homologous landmarks. However, these individuals were still considered for the linear metric and qualitative analyses if the first sacral vertebra could be clearly identified. Specimens were excluded from the linear metric analysis if the first proper sacral element could not be clearly identified [see 44] or if erosion of the upper portion of the sacrum prevented accurate measurements. Erosion or damage, particularly of the caudal part of the sacrum, was also an exclusion criterion for the qualitative investigation (see also S1 Table in S1 Appendix).

Each of the three methodological approaches was tested using three different compositions of the sample. Initially, the entire sample was analysed to quantify global patterns of sexual dimorphism (GM: n = 107; linear metrics: n = 145; qualitative inspection: n = 154). Then we investigated population-specific trends in the Central Europeans from the Weisbach collection (GM: n = 39; linear metrics: n = 58; qualitative inspection: n = 58), which is rather homogeneous owing to its composition of mostly young military personnel. Finally, population-specific trends were also analysed in a more heterogeneous African subgroup consisting of the pooled West African, Khoesan and Pygmy subsamples (GM: n = 23; linear metrics: n = 35; qualitative inspection: n = 37). We performed an additional geometric morphometric analysis to focus on the shape variation between the geographic groups. For this, we excluded the Weisbach collection to avoid over-representation of the European subsample, yielding a total sample size of 68.

### Geometric morphometric analyses

**Fig 1:**
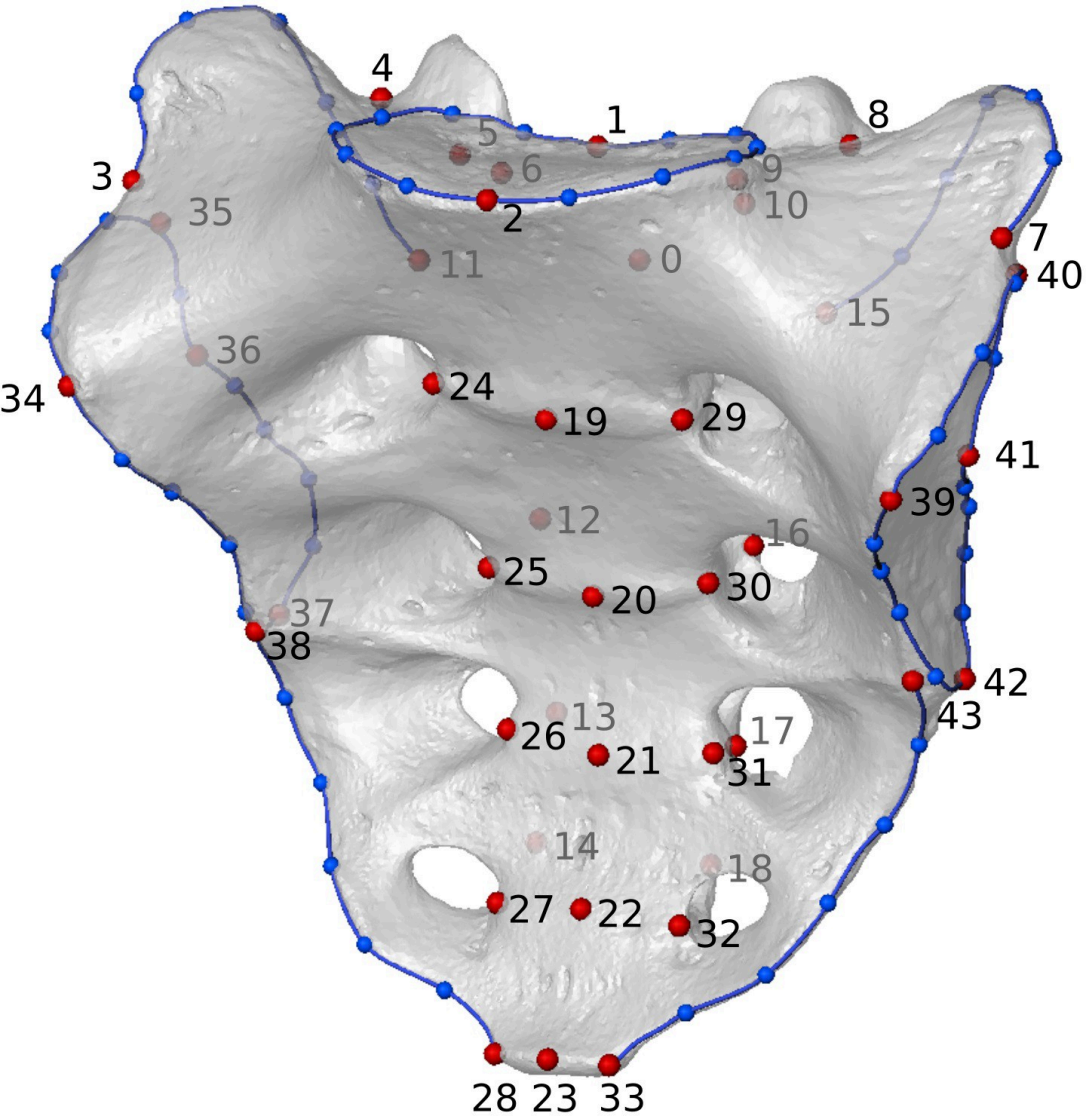
Landmark configuration representing the entire sacrum. The landmark configuration consists of 44 fixed landmarks (red dots) and 56 sliding semilandmarks (blue dots) on seven curves (i.e., superior articular surface, dorsal and lateral crests, and auricular surfaces; blue lines).

We used a landmark configuration consisting of 44 landmarks and 56 curve semilandmarks along seven curves [Fig 1 and S1 Table in S1 Appendix; see also ref. 33] to represent the morphology of the entire sacrum. Landmarks and curves were collected in Geomagic Design X (www.3dsystems.com). The semilandmarks were slid in the EVAN Toolbox (www.evan-society.org) by employing the bending energy technique [45]. All landmark configurations were standardized by scaling, rotating, and translating using a Generalized Procrustes analysis. Afterward, we performed a principal component analysis (PCA) on the Procrustes shape coordinates [see 46, 47 for a comprehensive overview of GM techniques].

For an additional analysis, shape space was converted to form space by augmenting the Procrustes shape coordinates by a column of the natural logarithm of centroid size (lnCS) [48, 49]. To test whether size was a reliable predictor of sex, we performed an independent t-test on the lnCS. Additionally, different subsets of the landmark and semilandmark configuration were analysed to explore the geographical and sex-related variation of different regions of the sacrum in more detail. Specifically, we used configurations representing the auricular surface and the upper part of the sacrum (first sacral vertebra, first and second sacral vertebrae, and only the corpus of the first sacral vertebra), as well as a reduced configuration based on anatomical landmarks only.

We regressed the shape variables (dependent variables) against lnCS, sex, and geographic origin (three separate independent variables) in the EVAN toolbox to investigate the amount of shape variance accounted for by each of these independent variables. Moreover, interactions between the independent variables were investigated using Procrustes ANOVAs with the ‘advanced.procD.lm’ function in the R geomorph package [50, 51]. To further account for potential sex driven allometric trends within the sample, the protocol of Torres-Tamayo et al. [52] and their corresponding R script (available from https://osf.io/zrgbf/?view_only=ffd87b50e6744ca780d43a758403d05a) was implemented to isolate size effects. The latter analysis was performed by regressing the allometric residuals (dependent variable) on sex (independent variable) in binary form. Allometric shape changes were subsequently visualized using warped models based on the regression coefficients for lnCS. The statistical significance of the differences between the male and female group means was verified by a permutation test of the Procrustes distances (10,000 random permutations) and visualized using group mean difference deformations. Visualizations were generated in the EVAN toolbox.

### Linear metrics

On the digital models, we measured corpus width of the first sacral vertebra (CW; according to Martin and Saller [53, 54] measurement No. 19) and sacral width (SW; according to Martin and Saller [53, 54] measurement No. 5). This yielded the corresponding corporo-basal-index (CBI) of Fawcett [7] as

$$CBI=\frac{CW}{SW}\times 100,$$

which is similar to the base-wing-index of Kimura [11]. Further, we measured the depth of the corpus of the first sacral vertebra (CD; according to Martin and Saller [53, 54] measurement No. 18), which was used to calculate the superior surface area of the corpus of the first sacral vertebra (CW × CD) and the associated corpus area index (CAI) adapted from index I-5 of Flander [8], as

$$CAI=\frac{\sqrt{CW\times CD}}{SW}\times 100.$$

Differences in male and female group means were statistically evaluated via an independent t-test.

### Qualitative inspection

For the qualitative investigation, the 3D surface models were anonymized, and three investigators (V.A.K, C.F., M.H.) determined sex twice with at least two weeks between subsequent assessments. Each observer considered sacral gross morphology and focused on specific features such as coronal and sacral curvature, corpus-ala relationship, height to width proportion, and the expansion of the auricular area.

### Classification accuracy

Classification accuracy was reported separately for the complete sample, the European and the African subsamples. For the geometric morphometric approach, classification accuracy was assessed using linear discriminant analysis (LDA) in SPSS (www.ibm.com) with a leave-one-out cross-validation of the results. The LDA assumption of homogeneity of covariance matrices was tested using Box’s M Test and log determinants. To avoid the introduction of noise into these analyses, only shape space PC scores with a significant group mean difference between males and females were considered [33]. Each of the linear metric measurements was tested independently using LDAs. The qualitative inspection was validated using cross-tables. Specifically, the accuracy of the sex estimation was calculated according to the formula

$$accuracy=sensitivity\times prevalence+specificity\times \left(mathvariant="italic">1-prevalence\right),$$

i.e., the ratio of correctly classified males in our sample multiplied by the prevalence of males plus the ratio of correctly classified females multiplied by 1 minus the prevalence of males.

For a direct comparison of the discriminatory power of the different methods, Receiver Operator Characteristic (ROC) curves were computed. ROC curves are probability curves for binary classifications created by plotting true-positive against false-positive rates at various thresholds. The ROC curves were based on the group memberships predicted by the LDA, but, in contrast to the LDA, only data sets that were investigated with all three methods were taken into account for the ROC curves. This led to slightly larger sample sizes for the LDA than the ROCs. Accordingly, the corresponding classification accuracies are expected to differ slightly. Females were represented by positive values in the visualizations. Additionally, the AUCs (areas under the curve) were calculated and subsequently represented as a scale ranging from 0 to 1. This represents an aggregate measure of performance across the possible classification thresholds. As such, a model whose predictions are wrong in all instances would produce an AUC of 0, while one whose predictions are correct in all cases delivers an AUC of 1. The AUC is thereby equivalent to the probability that a randomly chosen positive instance is ranked higher than a randomly chosen negative instance, functioning statistically as a two-sample Wilcoxon rank-sum statistic. For comparability of the AUCs, the ROC curves were built including the specimens that could be investigated with all three different methods.

## Results

### Geometric morphometric analyses

**Fig 2:**
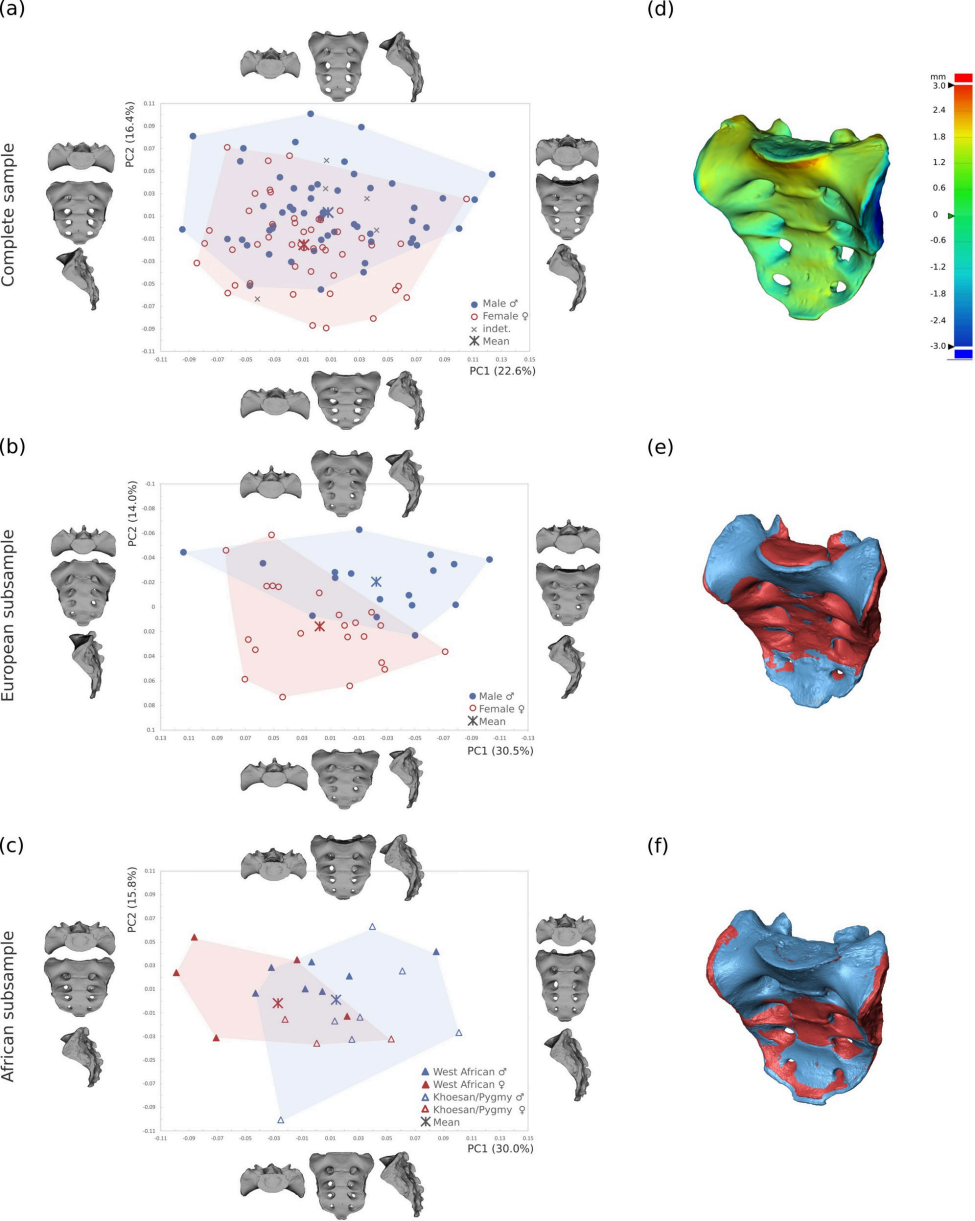
PCA plots of the Procrustes shape coordinates (PC1 vs. PC2). PCA plot of (a) the complete sample (n = 107), (b) the European subsample (n = 39), and (c) the African subsample (n = 23). The thin-plate-spline warps represent the real shape variation at the extremes of the ranges of variation. (d) Colour-coded representation of the morphology changes from the female into the male mean shape of the entire sample. Warm colours indicate a positive deviation, cold colours denote a negative deviation. Superimposed male (blue) and female (red) mean shapes in the (e) European and (f) African subsamples.

The GM analysis of the entire modern human sample showed extensive overlap between male and female sacra of the various populations (see Fig 2 and Table 3). The overlap between the sexes was particularly pronounced along PC1, with males slightly more variable than females. The shape changes along PC1 (explaining 22.6% of the total variance) reflected varying degrees of sagittal curvature. Along PC2 (16.4%), the overlap between the sexes was slightly less pronounced, still only the most extreme male and female sacral morphologies separated from the rest of the sample. Nevertheless, the mean shapes differed significantly (*p* < 0.01). The average female sacrum was shorter and broader, with less anterior protrusion of the promontory, a deeper coronal curvature, and broader alae with respect to the corpus of the sacral vertebrae. Hence, the typical male sacrum was relatively longer and narrower compared to females, with a more anteriorly protruding promontory, elongated auricular surface, and a broader corpus of the first sacral vertebra with respect to the mediolaterally narrow alae (Fig 2). PC3 was associated with varying craniocaudal orientation of the sacral alae and sacral canal expansion and, like the subsequent PCs, did not reflect sexual variation. We performed the same analysis using only the fixed landmarks and obtained similar outcomes, thus these results were not reported. As expected, in form space, size was the dominant factor along PC1. However, the separation between males and females did not improve compared to shape space analyses (S1 Fig in S1 Appendix).

**Table 3:** Percentages of principal component (PC) explained variances in both shape and form space, and percentages of accounted variance by selected independent variables yielded by the multivariate regressions (natural logarithm of centroid size lnCS, sex, and geographic origin).

PCA in shape space	PC1	PC2	PC3
Complete sample	22.6	16.4	8.9
Europeans	30.5	14.1	8.4
Africans	30.0	15.8	9.3
PCA in form space	PC1	PC2	PC3
Complete sample	40.3	13.3	9.8
Europeans	36.8	19.3	8.5
Africans	51.3	15.6	6.2
Multivariate regression of shape variables	lnCS	Sex	Geographic origin
Complete sample	1.7	2.2	3.7
Europeans	3.6	6.6	-
Africans	6.5	9.1	-

Abbreviation: PC, principal component; lnCS, natural logarithm of centroid size.

The GM analysis of the European subsample showed the same general pattern of shape variation observed for the complete sample along each of the PCs (Fig 2 and S1 video; see Table 3 for the percentages of the variance explained). Nevertheless, based on the corpus-to-alae relationship, there was a more apparent separation of the sexes along the angle bisector between PC1 and PC2. In contrast to the analysis performed on the full sample, males and females showed little overlap along PC1 in form space (S1 Fig in S1 Appendix).

In the relatively small African subsample (n = 23), the distinction between the sexes occurred along PC1 instead of PC2, but still according to the same pattern of sex variation as detected in the analyses of the full sample and the European subsample (Fig 2). The Khoesan and Pygmies expressed little sagittal curvature, which was reflected along PC2. In form space, the Khoesan and Pygmies separated from the West African specimens on PC1 as a consequence of the body size differences between the groups. The sample size was too small to confidently assess separation between the sexes using PC2 (S1c Fig in S1 Appendix).

**Fig 3:**
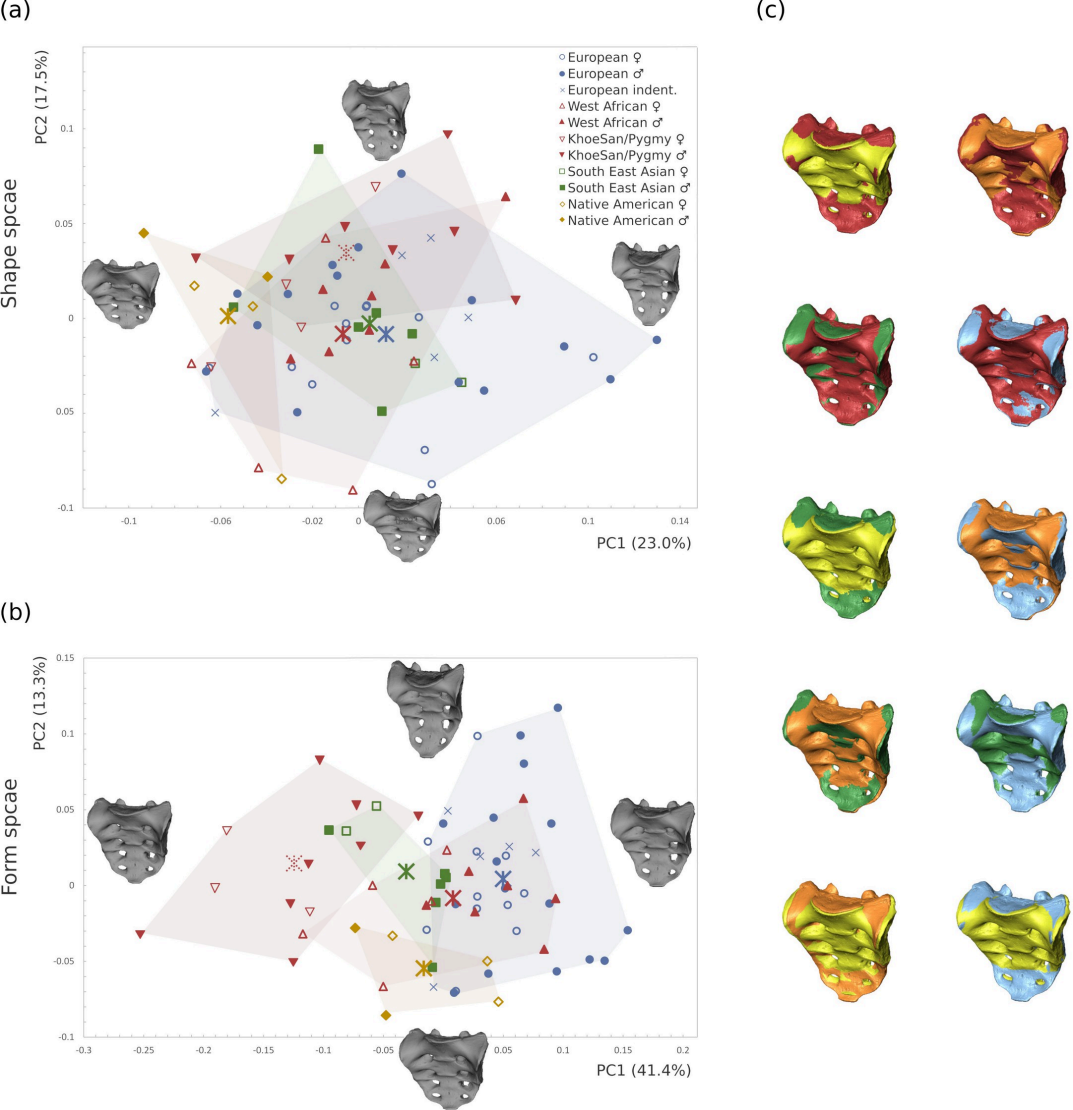
PCA plot of the sample without central europeans (n = 68). The Central European specimens from the Weisbach collection were excluded to avoid overrepresentation of the European subgroup. The thin-plate-spline warps show the shapes at the extremes of the axes in (a) shape space (b) form space. (c) Pairwise comparisons between the mean shapes of the different subgroups (Europeans = blue, West Africans = red, Khoesan and Pygmies = orange, South East Asians = green, Native Americans = yellow).

Population-specific shape variation was not detected in the sacrum since all populations overlapped considerably in shape space (Fig 3A and 3C). In form space (Fig 3B), size became the most important factor leading to the separation of Khoesan and Pygmies from the other groups. Form changes were extensive and concerned multiple aspects of the sacrum. The sacral curvature was flatter in the Pygmies and Khoesans as well as in the Native Americans from Tierra del Fuego, and most pronounced in the Europeans, with the remaining groups being intermediate. Sacral curvature, therefore, seemed to correlate mainly with body size. Variation at the the sacral canal was also noted, with Pygmies and Khoesans showing a relatively spacious sacral canal compared to the European sample.

The analyses of the landmark subsets representing the auricular surface and the upper part of the sacrum showed that in the complete sample neither the first vertebra nor the combined first and second sacral vertebrae differentiated between the sexes or geographic categories for any PC (S2 Fig and S3 Table in S1 Appendix). Similarly, the shape of the auricular surface was not sexually dimorphic. Shape changes along PC1 were associated with the relative width of the inferior leg of the auricular surface. Along PC2, we observed changes in the relative length of the superior leg of the auriculum (S2 Fig in S1 Appendix). Similarly, the Procrustes fit on the landmarks representing the corpus did not yield a better separation.

**Fig 4:**
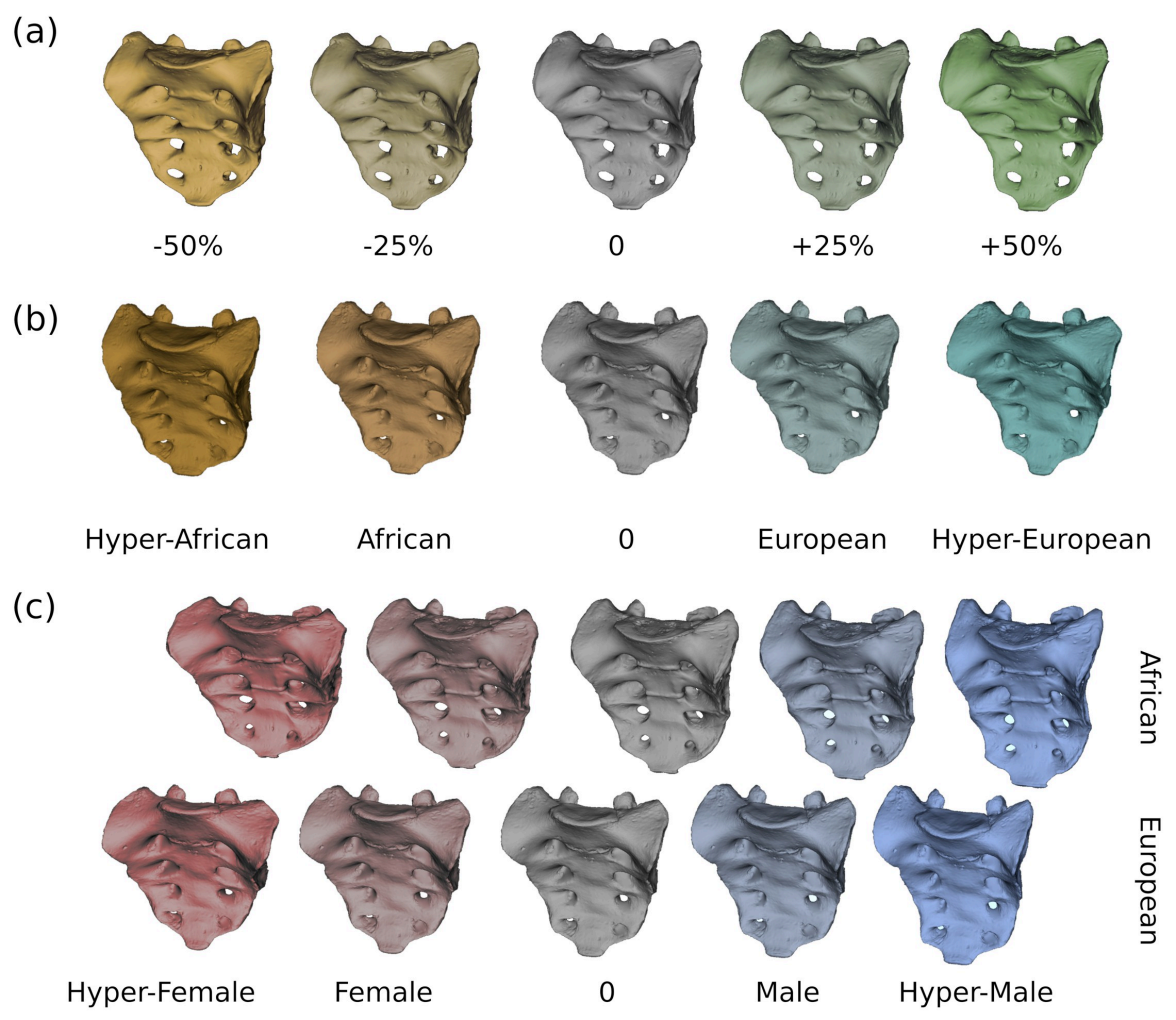
Variation of the sacrum shape depending on size, geographic region, and sex. (a) Multivariate regression against lnCS. The warped models are based on the regression coefficients and are shown, from left to right, at 50% and 25% smaller than average size, average size, and at 25% and 50% larger than average size. (b) Geographic group mean difference deformations between African and European subsamples (from left to right: exaggerated-African, African mean, general mean, European mean, exaggerated-European); (c) group mean difference deformations between females and males (left to right shapes: exaggerated-female, female mean, general mean, male mean, exaggerated-male). Note that the mean shapes used for group mean differences in (b) and (c) were not corrected for allometry as this is part of the variation in these groups.

The results of the multivariate regression analyses for the three independent variables, including sacrum size (lnCS), sex, and geographic origin are reported separately in Table 3. These variables produced no significant interaction terms when the entire sample was considered (S4 Table in S1 Appendix). The fraction of variance explained by allometry was low for the complete data set (1.6%) and not statistically supported (p = 0.073). However, size accounted for 3.4% and 3.2% of the explained variance in the European and African subsamples, respectively, though a statistically significant allometric trend was only observed for the European subset (p<0.05). Larger sacra tended to be more curved sagittally, whereas smaller ones were flatter (Fig 4A). Size (lnCS) differed significantly between the sexes in the Europeans (*p* < 0.001) and in the Africans (*p<*0.05), but not in the complete sample (*p* = 0.296) (S4 Table in S1 Appendix).

The sacrum shape as predicted by the regression analysis was sagittally and coronally straight for the Africans and more curved for the Europeans. Other shape changes affected the corpus to alae proportion, the expansion of the sacral canal, and the distance between the articular processes (see Fig 4B and S2 Video for visualization of group mean deformations).

Sex-related shape variation after correction for allometry accounted for a total variation of 2.2% in the complete sample, 6.6% in the European and 9.1% in the African sample. Shape changes followed the expected trajectories for the corpus to alae proportions and general width-to-length proportions in both the European and African subsamples (see Fig 4C and S3 Video for visualizations of group mean deformation).

### Linear metrics

**Fig 5:**
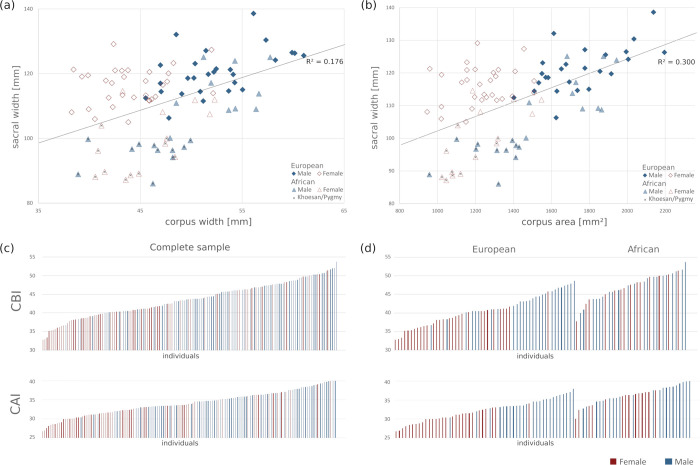
Variation of the linear metrics in the European and African subsets. (a) Scatterplot of sacral width (mm) and corpus width (mm); note the minimal overlap of females (red) and males (blue) in the European subset and the strong overlap in the African subset for corpus width. (b) Scatterplot of corpus depth (mm) and corpus width (mm) showing high correlation. (c) Fequency distribution of the corporo-basal index (CBI) and corpus-area index (CAI) for the complete sample. (d) Frequency distribution of the CBI and CAI for the European and the African subsamples; note the differences in the European (high discriminative power) and the African subsets (no discriminative power for CBI and moderate discriminative power for CAI).

The linear measurements and associated univariate statistics can be found in Table 4. All measurements differed significantly between the sexes in all three samples (*p* < 0.05), except for sacral width, which was significantly sexually dimorphic in the European subsample only (*p* = 0.013). Corpus depth and corpus area differed the most between sexes, maintaining consistent statistical significance across analyses. The corporo-basal index was significantly different in the complete sample and the European subsample (both *p* < 0.01), but not for the Africans (*p* = 0.600). Direct comparisons between the European and African data sets are visualized in Fig 5. Sacral width correlated moderately with corpus width (R = 0.42) and with corpus area (R = 0.55). Corpus width and corpus depth were strongly correlated (R = 0.70). Absolute sacral width and corpus width values as well as the CBI are reported in Table 5 for all individual and geographic groups in comparison to previously published data.

**Table 4:** Univariate statistics of the linear measurements.

Measurements		Complete sample		Europeans		Africans	
		F (n = 68)	M (n = 77)	F (n = 31)	M (n = 27)	F (n = 14)	M (n = 21)
Sacral width [mm]	Mean	112.3	113.7	116.1	120.5	99.0	104.5
	Standard deviation	10.0	10.8	5.8	7.0	10.1	11.5
	Minimum	87.2	86.1	105.0	106.3	87.2	86.1
	Maximum	133.4	138.6	129.1	138.6	114.6	125.1
	Average difference	1.4		4.4		5.5	
t-test	Significance (Cohens *D*)	*p* = 0.404		*p* = 0.013* (0.66)		*p* = 0.154	
Corpus width [mm]	Mean	46.1	51.5	44.1	52.4	46.0	49.3
	Standard deviation	4.3	4.7	3.5	4.3	3.9	5.1
	Minimum	38.3	38.9	38.3	45.5	40.6	38.9
	Maximum	56.9	62.0	52.2	61.0	52.3	57.8
	Average difference	5.4		8.3		3.3	
t-test	Significance (Cohens *D*)	*p* < 0.001** (1.03)		*p* < 0.001** (1.46)		*p* = 0.048* (0.66)	
Corpus depth [mm]	Mean	28.3	31.8	27.7	33.2	26.4	30.2
	Standard deviation	2.9	2.8	2.1	2.1	1.9	2.9
	Minimum	23.9	24.6	23.9	30.0	24.0	24.6
	Maximum	37.3	38.1	31.7	38.1	30.7	35.8
	Average difference	3.5		5.5		3.8	
t-test	Significance (Cohens *D*)	*p* < 0.001** (1.05)		*p* < 0.001** (1.60)		*p* < 0.001** (1.23)	
CBI [unitless]	Mean	41.3	45.4	38.0	43.6	46.7	47.3
	Standard deviation	4.6	3.8	3.2	3.1	3.6	3.7
	Minimum	31.7	36.7	31.7	36.7	37.8	40.0
	Maximum	51.4	53.7	45.8	48.6	51.4	53.7
	Average difference	4.1		5.6		0.6	
t-test	Significance (Cohens *D*)	*p* < 0.001** (0.89)		*p* < 0.001** (1.35)		*p* = 0.600	
CAI [unitless]	Mean	32.4	35.7	30.2	34.5	35.4	37.0
	Standard deviation	3.0	2.5	2.0	1.7	2.2	2.4
	Minimum	25.5	30.4	25.5	30.4	30.0	32.8
	Maximum	40.0	42.2	34.0	37.9	37.6	42.2
	Average difference	3.3		4.3		1.6	
t-test	Significance (Cohens *D*)	*p* < 0.001** (1.03)		*p* < 0.001** (1.50)		*p* = 0.048* (0.64)	
Corpus area [mm²]	Mean	1300	1650	1230	1750	1200	1500
	Standard deviation	235	265	150	200	170	275
	Minimum	940	960	940	1400	1000	950
	Maximum	2000	2200	1500	2200	1500	1900
	Average difference	350		520		300	
t-test	Significance (Cohens *D*)	*p* < 0.001** (1.17)		*p* < 0.001** (1.65)		*p* < 0.001** (1.10)	

*significant (*p* < 0.05),

**highly significant (*p* < 0.01).

Abbreviation: M, male; F, female; CBI, corporo-basal index; CAI, corpus area index.

**Table 5:** Absolute measurements of sacral width, corpus width, and the associated corporo-basal index in this study compared to previously published mean values.

Publication	Population	n (F;M)	Sacral width (mm)		Corpus width (mm)		Corporo-basal index	
			F	M	F	M	F	M
Present study	European (incl. Weisbach)	95 (48;47)	116.3	118.6	45.9**	52.6**	39.51**	44.42**
	South East Asian	9 (2;7)^§^	98.9	110.4	41.2	50.6	41.60	45.94
	West African	16 (6;10) ^§^	106.9	114.3	48.1	53.3	45.23	46.75
	Khoesan-Pygmy	20 (9;11) ^§^	92.4	95.6	44.3	45.7	48.01	47.89
	Native American	6 (4;2) ^§^	117.3	107.0	51.5	52.1	43.82	48.77
Benazzi, Maestri [24]	Italian	111 (56;55)	112.3	112.3	43.9**	50.0**	39.09^‡^	44.52^‡^
Davivongs [37]	Australian Aboriginal	100 (50;50)	101.2	99.92	44.10**	47.4**	43.62**	47.42**
Etli, Asirdizer [25]	Turkish	480 (240;240)	117.3	118.2	52.5*	55.5*	44.9*	47.1*
Flander [8]	European American	100 (50;50)	117.6	116.4	46.6**	52.8**	39.64**	45.42**
	African American	100 (50;50)	111.4	111.1	47.4**	54.5**	42.66**	49.02**
Fawcett [7]	European American	213 (79;134)	NA	NA	NA	NA	40.5	45.0
	African American	196 (88;108)	NA	NA	NA	NA	42.4	48.6
Kimura [11]^†^	Japanese	103 (51;52)	116.4*	114.4*	45.1*	50.0*	38.75*	43.71*
	European American	100 (50;50)	124.6*	122.9*	43.6*	48.9*	36.00*	39.79*
	African American	97 (48;49)	118.6*	114.4*	43.6*	48.8*	36.76*	42.66*
Mahato [55]	Indian	78 (female only)	108.7	NA	40.3	NA	37.07^‡^	NA
Mishra, Singh [56]	Indian	116 (42;74)	105.8	105.3	42.8**	49.1**	40.50**	46.50**
Raghavendra, Reddy [57]	Indian	200 (100;100)	99.9	101.6	37.8**	41.2**	37.93**	40.57**
Sachdeva, Singla [58]	North Indian	50 (10;40)	101.7	103.1	45.5	47.6	43.84	43.42
Steyn and İşcan [34]	Greek	192 (95;97)	115.6	116.6	47.1**	49.5**	40.74^‡^	42.45^‡^
Strádalová [16]	Chech	128 (56;72)	114.9	117.3	47.3	51.4	41.17*	43.95*
Tague [35]	European American	97 (44;52)	116.3	117.5	45.7**	53.1**	39.29^‡^	45.19^‡^
	African American	101 (50;51)	110.8	111.6	46.2**	53.2**	41.69^‡^	47.67^‡^
Torimitsu, Makino [29]	Japanese	230 (115;115)	115.2	117.0	52.3**	57.4**	45.40^‡^	49.06^‡^
Zech, Hatch [30]	Swiss	95 (46;49)	110.1	115.0	46.5*	53.5*	42.23^‡^	46.52^‡^

*significant (*p* < 0.05);

** highly significant (*p* < 0.01).

† Kimura (1982) used Base-Wing-Index: Width of the Base = corpus width, sacral width = width of the base + 2x width of the wing; corporo-basal index recalculated.

‡ corporo-basal index calculated from given measurements.

§ due to the small sample size not tested for significance.

Abbreviations: F, females; M, males.

### Qualitative inspection

Although all three investigators focused on the same features, the qualitative investigation showed a high inter-observer discrepancy of up to 25%. Intra-observer reliability was about 84% for all investigators in all samples. Precision (sum of true positives divided by sum of predicted positives; positive condition equals females) varied up to 30 percent points, being most accurate in the European subset (0.8) and lowest in the African subset (0.5), varying little between the investigators. Sensitivity (sum of true positives divided by sum of true positives plus false negatives) varied by 15 percent points between the samples and 30 percent points between the investigators. Specificity (sum of true negatives divided by sum of true negatives plus false positives) varied by 12 percent points between the samples and 18 percent points between the investigators.

### Classification accuracy

#### Intra-method classification accuracy

*Geometric morphometric analyses*. Not all PCs of the GM analyses were informative with regard to sex determination. Instead, additional factors other than those related to sexual dimorphism were captured when the entire sacral shape was investigated. For example, in the European subsample, only PC1, PC2 and PC5 were diagnostic for sex classification purposes in shape space and PC1, PC3 and PC6 in form space.

Thus, the classification accuracy for the GM approach was not exactly comparable between the three investigated samples because different PC scores were considered. The cross-validated mean classification was 73% for the complete sample, 87% for the European subsample, and 64% for the African subsample. The lnCS yielded weaker results (59% for the complete sample, 80% for the European subsample, and 60% for the African subsample). Combining shape variables with a measure of size (lnCS) yielded the best results.

*Linear metrics*. Among all linear metrics, absolute corpus depth was the most powerful discriminator with a classification accuracy of 78% for the complete sample, 93% for the European, and 79% for the African subsample. Corpus width was likewise quite accurate, with 74% for the complete sample, 86% for the European, and 66% for the African subsample. Therefore, corpus area and the corpus-area index performed considerably well. In contrast, the corporo-basal index showed less discriminative power. Sacral width was the weakest linear discriminator with classification accuracies of 54%, 62% and 53%, respectively, for the complete, the European and the African subsamples.

*Qualitative inspection*. For the qualitative approach, accuracy was highest in the European subsample (75%) and lower in the complete sample (69%) and the African subset (62%), with high levels of inter-observer discrepancy (25%).

#### Inter-method classification accuracy

The classification accuracies to male or female sacrum for the various parameters are shown in Table 6. Corpus depth was the single most powerful discriminator for all samples. Corpus width and the GM approach also classified reliably across all samples. Sacral width constantly yielded the weakest discriminatory power. Qualitative inspection produced inconsistent results, depending on the sample and investigator, although, overall, classification was more accurate for the European subsample.

**Table 6:** Percentages of cross-validated correct classifications.

Geometric morphometrics	Complete (n = 101)		European (n = 39)		African (n = 22)	
	F	M	F	M	F	M
PC scores shape space (significant PCs)	76.6	70.4	86.4	88.2.	62.5	64.3
lnCS	55.3	63.0	77.3	82.4	62.5	57.1
PC scores shape space & lnCS	78.7	74.1	95.5	88.2	62.5	71.4
Linear data	Complete (n = 139)		European (n = 58)		African (n = 37)	
	F	M	F	M	F	M
Sacral width (SW, mm)	49.2	59.5	61.3	63.0	64.3	42.9
Corpus width (CW, mm)	76.9	71.6	90.3	81.5	64.3	66.7
Corpus depth (CD, mm)	80.6	75.3	90.0	96.0	85.7	71.4
Corporo-basal index (CW/SWx100)	72.3	73.0	77.4	77.8	42.9	57.1
Corpus-area index (√CWxCD/SWx100)	76.1	71.2	83.3	92.0	42.9	57.1
Corpus area (CWxCD)	82.1	72.6	93.3	96.0	78.6	66.7
Qualitative investigation	Complete (n = 140)		European (n = 58)		African (n = 37)	
	F	M	F	M	F	M
Researcher 1	77.7	70.7	88.7	72.2	75.0	71.4
Researcher 2	60.0	74.0	59.7	90.7	32.1	76.2
Researcher 3	72.1	58.7	64.5	74.1	60.7	54.8

Abbreviations: PC, principal component; lnCS, natural logarithm of centroid size; F, female; M, male.

**Fig 6:**
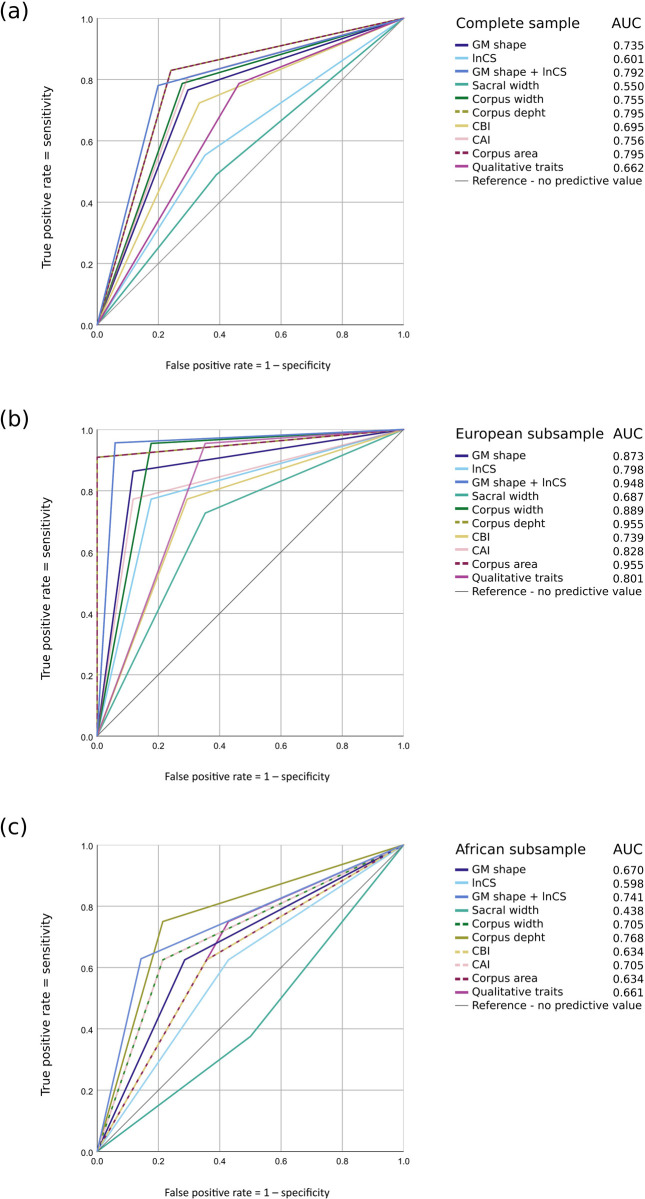
ROC curves based on predicted group membership from linear discriminant analyses for all classification methods reporting the area under the curves (AUCs). (a) ROC curves for the complete sample (n = 101), (b) the European subset (n = 39) and (c) the African subset (n = 22). Corpus depth was the single most discriminative measurement while sacrum width was the weakest for all samples. Note that dotted lines represent similar thresholds for two parameters (i.e., corpus depth/corpus area in a and b; corpus width/CAI and corpus area/CBI in c).

The results of the performance assessment for the three classification approaches, including the ROC curves and the corresponding AUCs are reported in Fig 6. Females were represented by positive values in the visualizations. The slightly different sample sizes used for the ROCs with respect to the LDA (reported in Table 6) resulted in slightly different corresponding classification accuracies. Based on the AUCs calculated from the ROC categorizations, none of our approaches seemed to be a suitable method for the classification of the complete data set (AUCs < 0.8). Conversely, corpus depth and area performed well in classifying specimens of European ancestry (AUCs > 0.9). Corpus width, corpus area index, PC scores, and qualitative traits were also classified comparatively well in Europeans (AUCs between 0.8 and 0.9). Except for corpus depth, all approaches were deemed poor classifiers for the African subset (AUCs < 0.7).

## Discussion

Our study focused on the investigation of sexual dimorphism of the sacrum in a geographically heterogeneous modern human sample to quantify associated shape changes and detect possible population specific patterns of variation related to sex. We thereby compared the performance of traditional qualitative and metric approaches with that of landmark-based geometric morphometrics.

The shape differences between the sexes showed patterns widely recognized in the literature [e.g., 28, 37]. Namely, the mean female sacrum was relatively broad and short, while males typically possessed a relatively long and narrow sacrum. However, overall shape did not prove sufficient to reliably differentiate male from female individuals. In this study, the quantitative approaches were found to be more powerful than the qualitative investigation. The discriminatory power of the quantitative approaches was of comparable magnitude, although certain measures performed better than others in all of the investigated samples. Thus, corpus width and corpus depth (and their derivates) were the most powerful discriminators, even outperforming geometric morphometric shape analyses. Their discriminatory power was only matched by adding a measure of size, such as lnCS, to the shape coordinates. We explain this outcome with the high morphological variation of both sexes, resulting in a wide range of overlapping morphologies. The inclusion of additional, potentially sexually dimorphic, discrete traits of the sacrum (e.g., sacral preauricular extensions and sacral preauricular notches) would not likely alter our findings. In fact, these features have been associated with multiple pregnancies and births before age 25 [59]. However, their rarity and their discrete nature makes it difficult to capture them with any of the investigated methods.

As expected, the degree of morphological variation of the sacrum is more pronounced with increasing geographical heterogeneity. However, all methods showed much lower classification accuracies for the African subset. This can either be explained as an artifact of our limited sample size or by the fact that most methods have traditionally been described based on populations of European origin and thereby do not perform well in other populations. Although metric parameters classified the sexes equally well in African Americans and European Americans in the studies of Flander [8], Kimura [11], and Rusk and Ousley [15], the inclusion of Khoesan and Pygmies in our African sample might explain our different outcome. The effect of body size on sacrum shape in Khoesan and Pygmies suggests caution and it might therefore be better to consider small-bodied popultions as separate groups in future works.

Group mean shape deformation showed that our African and European subsets expressed variable degrees of sagittal sacral curvature (Fig 4). Sacral curvature was more pronounced in the Europeans than in the rest of the sample and stronger in males than in females. This finding is supported by Flander [8], who reported similar differences between European Americans and African Americans, although she suggested that the deeply curved sacrum of European Americans could relate to osteomalacia or rickets and thereby be a reflection of malnutrition. In contrast, Plochocki [13] hypothesized that the shallower sagittal curvature of the female sacrum might represent an obstetrical adaptation to enlarge the pelvic outlet. However, radiological-obstetrical studies suggested that a flat sacrum compromises fetal head rotation and is associated with an increased incidence of operative deliveries [60, 61]. Another explanation for sacral curvature variation was provided by Abitbol [62], who suggested that it might be related to biomechanical factors associated with supine sleeping posture and differing effects of bipedal locomotion in individuals with varied body sizes, and is thus unrelated to obstetrical adaptation [see also 63]. Accordingly, the generally larger body size of males might lead to relatively greater tension of the sacrospinous and sacrotuberous ligaments, causing the sexual dimorphism in the sagittal curvature of the sacrum. Therefore, the flatter sacrum of Khoesan and Pygmies compared to Europeans could, in part, be explained by their smaller body size.

However, sacrum size alone (as represented by the natural logarithm of centroid size) was a weak classifier in our analyses, a finding that echoes the results of Rusk and Ousley [15]. Likewise, sacral width was a poor classifier in all samples, likely because similar absolute sacral widths are reached in males and females by different proportions of the corpus and the alae. Males possess mediolaterally narrower alae with a relatively broader corpus of the first sacral vertebra, whereas females show broader alae with respect to a smaller corpus. Tague [35] interpreted the broad female alae (i.e., costal process length) as the strongest obstetrical adaptation in the bony pelvis, as it increases the dimensions of the female birth canal, together with the elevation of the auricular surface [64]. The notion that obstetrical selection is indeed shaping sacrum morphology is further supported by the observation that women with relatively larger heads tend to give birth to children with large heads, and thus tend to have a sacrum that leaves more space in the birth canal [65].

Ultimately, overall size and width of the sacrum are less discriminative between the sexes than corpus-to-alae proportions. Nevertheless, the analyses of the landmark subsets on the first and second sacral vertebrae did not show better discrimination between the sexes than the landmark configuration capturing the entire sacrum. In fact, simple linear measurements on the corpus of the first sacral vertebra like corpus breadth, depth, and their derivatives, proved to be more diagnostic than any of the landmark- and semilandmark-based geometric morphometric approaches, although they only partially represented the sacrum. Similarly, linear metrics describing overall cranial size have been suggested to provide a simple option for preliminary classifications of age and sex, while the analysis of form space was particularly useful to increase confidence in group assessment [66]. The significant differences between the sexes and the high classification accuracy of the corpus of the first sacral vertebra are again most likely linked to overall body size and the need for efficient weight transmission from the upper to the lower body.

Another explanation for the relatively low obstetrical selection pressure on sacrum morphology may be the hormonally mediated increased sacroiliac joint mobility during birth. Thus, the sacroiliac joint provides a firm connection with only limited mobility between the sacrum and the hipbone in the non-pregnant condition [67–69], while during birth the dimensions of the pelvic outlet can be enlarged by up to 2 cm via nutation, i.e. backwards rotation of the apex of the sacrum, thereby alleviating some of the obstetric constraints on the bony sacrum [70–73]. Biomechanical constraints related to bipedal locomotion might therefore dominate in the sacrum over reproductive selection pressure. Similarly, mediolateral width and anteroposterior depth of the pelvis might be more constrained by bipedal locomotion or factors like thermoregulation rather than obstetrics [see Discussion in 74]. The sacroiliac joints might also buffer the biomechanical pressure of weight transmission during bipedal locomotion, a hypothesis supported by the lack of sufficient evidence for sex-based or geographically related differences in the shape of the auricular surface noted in this study.

The sacrum is a highly variable bone, and our results suggest that factors such as body size are important determinants of morphological variation in addition to sex, particularly when considering small-bodied populations such as the Khoesan and Pygmies. Future work is therefore needed to further disentangle the adaptive and neutral evolutionary processes shaping sacral morphology with careful consideration given to influential factors like geography, sex and body size.

## Supporting information

S1 Appendix Appendix with S1, S2 Figs and S1 to S4 Tables. (DOCX)

S1 Video (GIF)

S2 Video (GIF)

S3 Video (GIF)

S1 File (TXT)
