# Supplementary material for: Sex classification using the human sacrum: Geometric morphometrics versus conventional approaches
Source: PLoS One. 2022 Apr 6;17(4):e0264770. doi: 10.1371/journal.pone.0264770 (PMC8986015; doi:10.1371/journal.pone.0264770)
Supplement: S1 Appendix — (DOCX) [file pone.0264770.s001.docx]

**S1 Appendix**

Sex classification using the human sacrum: Geometric morphometrics versus conventional approaches

Viktoria A. Krenn^1,2*^, Cinzia Fornai^1,2 3^, Nicole M. Webb^1,4,5^, Martin Haeusler^1^

^1^ Institute of Evolutionary Medicine, University of Zurich, Switzerland

^2^ Department of Evolutionary Anthropology, University of Vienna, Austria

^3^ Vienna School of Interdisciplinary Dentistry, Klosterneuburg, Austria

^4^ Department of Palaeoanthropology, Senckenberg Research Institute and Natural History Museum Frankfurt, Germany

^5^ Institute of Archaeological Sciences, Senckenberg Centre for Human Evolution and Palaeo­environment, Eberhard Karls University of Tübingen, Germany

* Corresponding author

Email: [viktoria.krenn@iem.uzh.ch](mailto:viktoria.krenn@iem.uzh.ch)





**Fig** **S1** – PCA plot for PC1 and PC2 of the entire sacrum landmark configuration in form space

(a) complete sample, (b) European subsample and (c) African subsample


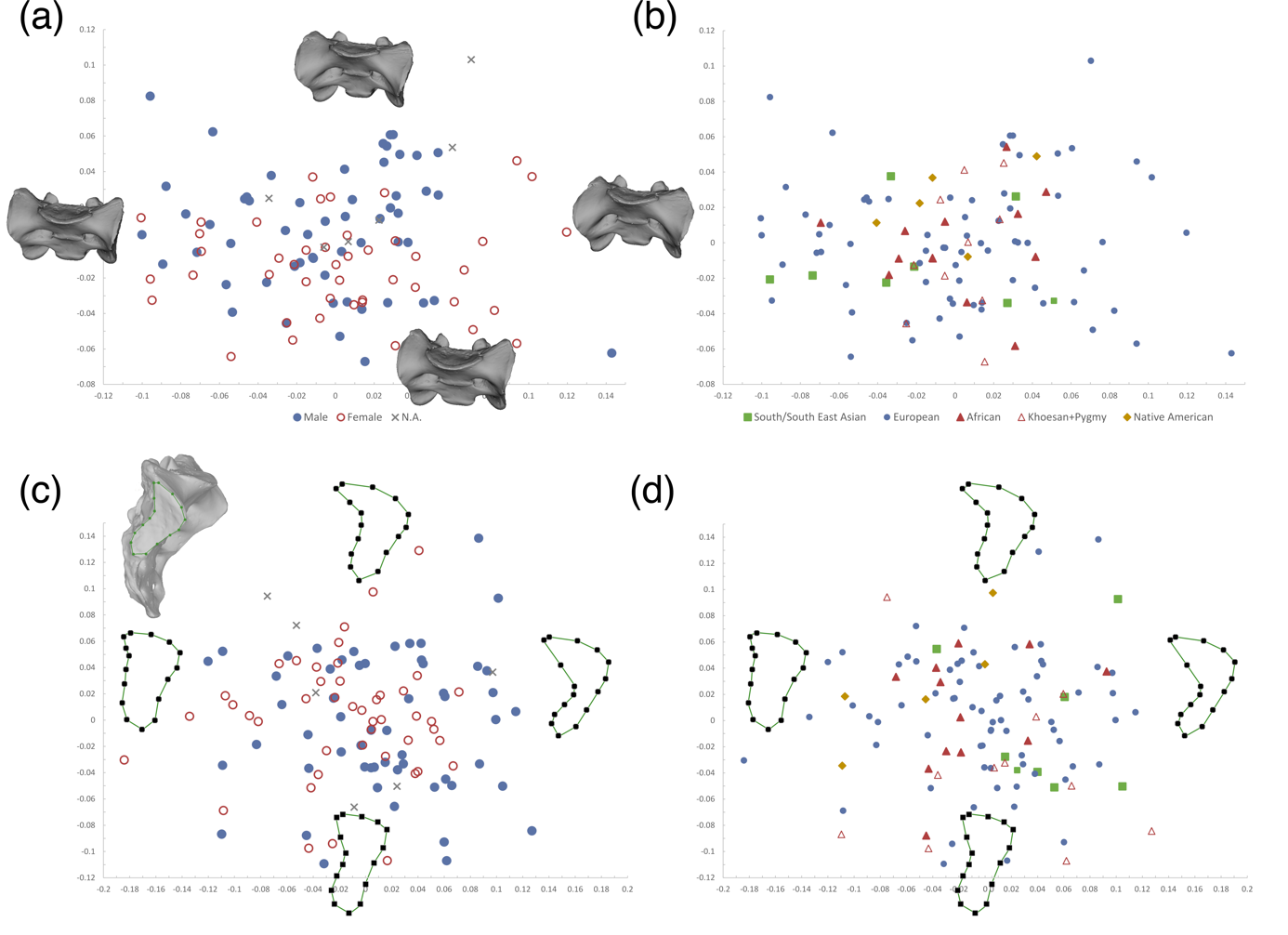


**Fig** **S2** – **PCA analyses of landmark subsets**. (a) The first sacral vertebra, colored by sex; (b) The first sacral vertebra, colored by geographic origin; (c) The auricular surface, colored by sex; (d) The auricular surface, colored by geographic origin.

**Table S1 – Sample composition (n=164) with details on repositories, performed analyses and reasons for exclusion from particular analyses**

| **Institution** | **Origin** | **Number** | **Sex** | **Age** | **Analyses** | **Exclusion criterion** |
| --- | --- | --- | --- | --- | --- | --- |
| AIMZ^1^ | South/South-East Asia | BIR_258 | m | N.A. | GM, LM, QI, SV |  |
| AIMZ | South/South-East Asia | IND_7542 | m | N.A. | GM, LM, QI, SV |  |
| AIMZ | South/South-East Asia | IND_7545 | m | N.A. | GM, LM, QI, SV |  |
| AIMZ | South/South-East Asia | IND_7546 | m | N.A. | GM, LM, QI, SV |  |
| AIMZ | South/South-East Asia | IND_7548 | f | N.A. | GM, LM, QI, SV |  |
| AIMZ | South/South-East Asia | IND_7550 | f | N.A. | GM, LM, QI, SV |  |
| AIMZ | South/South-East Asia | IND_7577 | m | N.A. | GM, LM, QI, SV |  |
| AIMZ | African | PAL_024 | m | N.A. | GM, LM, QI, SV |  |
| AIMZ | African | PAL_026 | m | N.A. | LM, QI, SV | segmentation anomaly |
| AIMZ | European | PAL_042 | m | N.A. | LM, QI, SV | segmentation anomaly |
| AIMZ | African | PAL_043 | m | N.A. | LM, QI, SV | segmentation anomaly |
| AIMZ | South/South-East Asia | PAL_045 | m | N.A. | GM, LM, QI, SV |  |
| AIMZ | African | PAL_057 | f | N.A. | GM, LM, QI, SV |  |
| AIMZ | European | PAL_077 | m | N.A. | LM, QI, SV | segmentation anomaly |
| AIMZ | African | PAL_098 | f | N.A. | GM, LM, QI, SV |  |
| AIMZ | African | PAL_103 | f | N.A. | GM, LM, QI, SV |  |
| AIMZ | African | PAL_126 | f | N.A. | GM, LM, QI, SV |  |
| AIMZ | European | PAL_131 | m | N.A. | LM, QI, SV | segmentation anomaly |
| AIMZ | African | PAL_153 | m | N.A. | LM, QI, SV | segmentation anomaly |
| AIMZ | South/South-East Asia | PAL_174 | m | N.A. | LM, QI, SV | segmentation anomaly |
| AIMZ | African | PAL_185 | m | N.A. | GM, LM, QI, SV |  |
| AIMZ | African | PAL_218 | m | N.A. | LM, QI, SV | segmentation anomaly |
| AIMZ | African | PAL_224 | m | N.A. | GM, LM, QI, SV |  |
| AIMZ | African | PAL_227 | m | N.A. | GM, LM, QI, SV |  |
| AIMZ | African | PAL_228 | f | N.A. | GM, LM, QI, SV |  |
| AIMZ | African | PAL_229 | m | N.A. | GM, LM, QI, SV |  |
| AIMZ | African | PAL_230 | m | N.A. | GM, LM, QI, SV |  |
| AIMZ | African | PAL_253 | m | N.A. | GM, LM, QI, SV |  |
| AKHV^2^ | European | AKH_001 | m | 27 | SV | segmentation anomaly |
| AKHV | European | AKH_002 | m | 33 | GM, LM, QI, SV |  |
| AKHV | European | AKH_003 | f | 44 | GM, LM, QI, SV |  |
| AKHV | European | AKH_006 | f | 32 | GM, LM, QI, SV |  |
| AKHV | European | AKH_008 | f | 38 | GM, LM, QI, SV |  |
| AKHV | European | AKH_013 | m | 34 | GM, LM, QI, SV |  |
| AKHV | European | AKH_015 | m | 35 | GM, LM, QI, SV |  |
| AKHV | European | AKH_1 | f | N.A. | GM, LM, QI, SV |  |
| DEAV^3^ | European | CSO_004 | f | N.A. | LM, QI, SV | segmentation anomaly |
| DEAV | European | CSO_021 | f | N.A. | GM, LM, QI, SV |  |
| DEAV | European | CSO_090 | m | N.A. | LM, QI, SV | segmentation anomaly |
| DEAV | European | CSO_155 | f | N.A. | GM, LM, QI, SV |  |
| DEAV | European | CSO_451 | m | N.A. | GM, LM, QI, SV |  |
| DEAV | European | CSO_484 | m | N.A. | GM, LM, QI, SV |  |
| DEAV | European | CSO_599 | f | N.A. | LM, QI, SV | erosion |
| DEAV | European | CSO_611 | m | N.A. | GM, LM, QI, SV |  |
| DEAV | European | CSO_627 | m | N.A. | GM, LM, QI, SV |  |
| DEAV | European | CSO_632 | N.A. | N.A. | SV | erosion |
| DEAV | European | CSO_642 | m | N.A. | SV | erosion |
| DEAV | European | KK_1 | N.A. | N.A. | GM, LM, QI, SV |  |
| DEAV | European | KK_2 | N.A. | N.A. | GM, LM, QI, SV |  |
| DEAV | European | KK_gw | N.A. | N.A. | GM, LM, QI, SV |  |
| DEAV | European | KK_P. Fl. M. 8 | N.A. | N.A. | GM, LM, QI, SV |  |
| DEAV | KhoeSan | SAN_008 | m | N.A. | GM, LM, QI, SV |  |
| DEAV | KhoeSan | SAN_012 | m | N.A. | GM, LM, QI, SV |  |
| DEAV | KhoeSan | SAN_061 | m | N.A. | GM, LM, QI, SV |  |
| DEAV | KhoeSan | SAN_068 | m | N.A. | GM, LM, QI, SV |  |
| DEAV | KhoeSan | SAN_074 | f | N.A. | GM, LM, QI, SV |  |
| DEAV | KhoeSan | SAN_075 | f | N.A. | GM, LM, QI, SV |  |
| DEAV | KhoeSan | SAN_085 | N.A. | N.A. | - | erosion |
| DEAV | KhoeSan | SAN_102 | N.A. | N.A. | GM, LM, QI, SV |  |
| DEAV | KhoeSan | SAN_104 | m | N.A. | LM, QI, SV | segmentation anomaly |
| DEAV | KhoeSan | SAN_109 | m | N.A. | GM, LM, QI, SV |  |
| DEAV | KhoeSan | SAN_110 | m | N.A. | SV | segmentation anomaly |
| DEAV | KhoeSan | SAN_128 | m | N.A. | GM, LM, QI, SV |  |
| IEMZ^4^ | European | B59 | N.A. | N.A. | GM, LM, QI, SV |  |
| IEMZ | European | G11 | m | 18 | - | erosion |
| IEMZ | European | G12 | m | N.A. | GM, LM, QI, SV |  |
| IEMZ | European | Ufenau 1968 | f | N.A. | GM, LM, QI, SV |  |
| MAEF^5^ | Native American | TDF_3116 | f | N.A. | GM, LM, QI, SV |  |
| MAEF | Native American | TDF_3119 | f | N.A. | LM, QI, SV | erosion |
| MAEF | Native American | TDF_3122 | m | N.A. | GM, LM, QI, SV |  |
| MAEF | Native American | TDF_3124 | f | N.A. | GM, LM, QI, SV |  |
| MAEF | Native American | TDF_3128 | f | N.A. | GM, LM, QI, SV |  |
| MAEF | Native American | TDF_3143 | m | N.A. | GM, LM, QI, SV |  |
| MNHN^6^ | Pygmy | PYG_09889 | f | N.A. | LM, QI, SV | erosion |
| MNHN | Pygmy | PYG_17761 | f | N.A. | LM, QI, SV |  |
| MNHN | Pygmy | PYG_17762 | m | N.A. | LM, QI, SV | erosion |
| MNHN | Pygmy | PYG_18449 | f | N.A. | LM, QI, SV | segmentation anomaly |
| MNHN | Pygmy | PYG_22257 | m | N.A. | LM, QI, SV |  |
| MNHN | Pygmy | PYG_23642 | m | N.A. | LM, QI, SV | segmentation anomaly |
| NHMV^7^ | European | WB_00667 | f | 28 | LM, QI, SV | erosion |
| NHMV | European | WB_00671 | f | N.A. | GM, LM, QI, SV |  |
| NHMV | European | WB_00694 | f | 22 | GM, LM, QI, SV |  |
| NHMV | European | WB_00696 | f | 23 | LM, QI, SV | erosion |
| NHMV | European | WB_02295 | m | 34 | LM, QI, SV | segmentation anomaly |
| NHMV | European | WB_02296 | m | 23 | LM, QI, SV | erosion |
| NHMV | European | WB_02297 | f | 35 | GM, LM, QI, SV |  |
| NHMV | European | WB_02298 | f | 25 | LM, QI, SV | segmentation anomaly |
| NHMV | European | WB_02300 | f | 25 | GM, LM, QI, SV |  |
| NHMV | European | WB_02301 | f | 30 | GM, LM, QI, SV |  |
| NHMV | European | WB_02302 | f | 35 | LM, QI, SV | erosion |
| NHMV | European | WB_02303 | f | 19 | GM, LM, QI, SV |  |
| NHMV | European | WB_02304 | f | 28 | GM, LM, QI, SV |  |
| NHMV | European | WB_02306 | f | 50 | GM, LM, QI, SV |  |
| NHMV | European | WB_02522 | m | 23 | LM, QI, SV | segmentation anomaly |
| NHMV | European | WB_02525 | m | 21 | LM, QI, SV | segmentation anomaly |
| NHMV | European | WB_02529 | m | 45 | LM, QI, SV | segmentation anomaly |
| NHMV | European | WB_02530 | m | 22 | GM, LM, QI, SV |  |
| NHMV | European | WB_02532 | m | 21 | GM, LM, QI, SV |  |
| NHMV | European | WB_02533 | m | 22 | LM, QI, SV | segmentation anomaly |
| NHMV | European | WB_02534 | m | 24 | LM, QI, SV | segmentation anomaly |
| NHMV | European | WB_02535 | m | 22 | GM, LM, QI, SV |  |
| NHMV | European | WB_02536 | m | 21 | GM, LM, QI, SV |  |
| NHMV | European | WB_02537 | m | 21 | GM, LM, QI, SV |  |
| NHMV | European | WB_02538 | m | 22 | LM, QI, SV | segmentation anomaly |
| NHMV | European | WB_02539 | m | 23 | GM, LM, QI, SV |  |
| NHMV | European | WB_02540 | m | 23 | GM, LM, QI, SV |  |
| NHMV | European | WB_02541 | m | 22 | GM, LM, QI, SV |  |
| NHMV | European | WB_02544 | m | N.A. | GM, LM, QI, SV |  |
| NHMV | European | WB_02545 | m | 58 | GM, LM, QI, SV |  |
| NHMV | European | WB_02548 | f | 20 | GM, LM, QI, SV |  |
| NHMV | European | WB_02550 | f | 20 | GM, LM, QI, SV |  |
| NHMV | European | WB_02552 | f | 18 | GM, LM, QI, SV |  |
| NHMV | European | WB_02553 | f | 77 | LM, QI | erosion |
| NHMV | European | WB_02554 | f | N.A. | GM, LM, QI, SV |  |
| NHMV | European | WB_02557 | f | 22 | GM, LM, QI, SV |  |
| NHMV | European | WB_02558 | f | 40 | GM, LM, QI, SV |  |
| NHMV | European | WB_02559 | f | 35 | GM, LM, QI, SV |  |
| NHMV | European | WB_02560 | f | 25 | LM, QI, SV | erosion |
| NHMV | European | WB_02561 | f | 28 | GM, LM, QI, SV |  |
| NHMV | European | WB_02562 | f | 20 | LM, QI, SV | segmentation anomaly |
| NHMV | European | WB_02564 | f | 68 | GM, LM, QI, SV |  |
| NHMV | European | WB_02567 | f | 26 | GM, LM, QI, SV |  |
| NHMV | European | WB_02568 | f | 32 | GM, LM, QI, SV |  |
| NHMV | European | WB_02569 | f | 23 | LM, QI | erosion |
| NHMV | European | WB_02570 | f | 19 | LM, QI, SV | segmentation anomaly |
| NHMV | European | WB_02572 | f | 28 | GM, LM, QI, SV |  |
| NHMV | European | WB_21584 | m | 21 | GM, LM, QI, SV |  |
| NHMV | European | WB_21591 | m | N.A. | LM, QI, SV | segmentation anomaly |
| NHMV | European | WB_21598 | m | 64 | GM, LM, QI, SV |  |
| NHMV | European | WB_21600 | m | 23 | GM, LM, QI, SV |  |
| NHMV | European | WB_21606 | m | N.A. | GM, LM, QI, SV |  |
| NHMV | European | WB_21607 | f | 30 | GM, LM, QI, SV |  |
| NHMV | European | WB_21608 | m | 21 | GM, LM, QI, SV |  |
| NHMV | European | WB_21609 | m | 26 | GM, LM, QI, SV |  |
| NHMV | European | WB_21610 | m | 23 | GM, LM, QI, SV |  |
| NHMV | European | WB_21611 | m | 24 | LM, QI, SV | segmentation anomaly |
| NHMV | European | WB_21620 | f | 25 | GM, LM, QI, SV |  |
| NMNH^8^ | European | SM_301623 | m | ~20 | GM, LM, QI, SV |  |
| NtNHMV^9^ | European | M_5998 | N.A. | N.A. | LM, QI, SV | segmentation anomaly |
| NtNHMV | European | NT_2563 | f | 22 | GM, LM, QI, SV |  |
| NtNHMV | European | STU_1 | f | N.A. | LM, QI, SV | segmentation anomaly |
| NtNHMV | European | STU_2 | m | N.A. | SV | segmentation anomaly, osteophytes |
| NtNHMV | European | STU_3 | f | N.A. | SV | Segmentation anomaly |
| PAAG^10^ | European | AIG_16 | f | 54 | GM, LM, QI, SV |  |
| PAAG | European | AIG_23 | m | 58 | GM, LM, QI, SV |  |
| PAAG | European | AIG_26 | m | 27 | GM, LM, QI, SV |  |
| PAAG | European | APP_04 | f | 31 | GM, LM, QI, SV |  |
| PAAG | European | APP_07 | f | 68 | SV | erosion |
| PAAG | European | ECH_02 | m | 50 | SV | erosion |
| PAAG | European | ECH_10 | m | 73 | GM, LM, QI, SV |  |
| PAAG | European | ECH_13 | m | 45 | LM, QI, SV | erosion |
| PAAG | European | ETA_08 | f | 36 | LM, QI, SV | segmentation anomaly |
| PAAG | European | LAU_03 | f | 44 | SV | segmentation anomaly |
| PAAG | European | LAU_12 | f | 46 | LM, QI | erosion |
| PAAG | European | LAU_13 | m | 18 | GM, LM, QI, SV |  |
| PAAG | European | LAU_33 | m | 59 | GM, LM, QI, SV |  |
| PAAG | European | LAU_47 | m | 41 | GM, LM, QI, SV |  |
| PAAG | European | MOI_02 | f | 51 | GM, LM, QI, SV |  |
| PAAG | European | MOI_11 | f | 18 | GM, LM, QI, SV |  |
| PAAG | Pygmy | PYG_01 | f | 30 | LM, QI, SV | segmentation anomaly |
| PAAG | Pygmy | PYG_02 | f | 30 | LM, QI, SV | segmentation anomaly |
| PAAG | Pygmy | PYG_03 | f | 35 | LM, QI, SV | segmentation anomaly |
| PAAG | Pygmy | PYG_04 | f | 55 | GM, LM, QI, SV |  |
| PAAG | Pygmy | PYG_05 | m | 65 | GM, LM, QI, SV |  |
| Abbreviations: N.A., not available; SV, number of sacral vertebrae  Performed analyses: GM, geometric morphometrics of sacra with five sacral elements; LM, linear metrics; QI, qualitative investigation  ^1^ AIMZ Anthropological Institute and Museum, University of Zurich, Zurich  ^2^ AKHV Department of Biomedical Imaging and Image-guided Therapy, Medical University of Vienna, Vienna  ^3^ DEAV Department of Evolutionary Anthropology, University of Vienna, Vienna  ^4^ IEMZ Institute of Evolutionary Medicine, University of Zurich, Zurich  ^5^ MAEF Museo di storia naturale, Firenze  ^6^ MNHN Musée de l’Homme, Paris  ^7^ NHMV Department of Anthropology, Natural History Museum, Vienna  ^8^ NMNH Smithsonian Institution, National Museum of Natural History, Washington  ^9^ NtNhM Pathological Collection Narrenturm, Natural History Museum, Vienna  ^10^ PAAG Laboratory of Prehistoric Archaeology and Anthropology, University of Geneva, Geneva | | | | | | |

| **Table S2 – Description of the landmarks and curves used to represent the sacrum in the GM analyses, from Krenn et al. (accepted)** | | |
| --- | --- | --- |
| Landmark | Location | Description |
| 0 | dorsal | Most cranial point of the median sacral crest |
| 1 | S1 corpus | Midsagittal point on posterior margin of superior sacral articular surface |
| 2 | S1 corpus | midsagittal point on anterior margin of the superior sacral articular surface (promontorium) |
| 3 | S1 right | Notch between the embryological transverse process and the costal process |
| 4 | S1 right | Superolateral base of the articular process |
| 5 | S1 right | Most lateral point of the sacral canal |
| 6 | S1 right | Most inferomedial point of the superior articular facet |
| 7 | S1 left | Notch between the embryological transverse process and the costal process |
| 8 | S1 left | Superolateral base of the articular process |
| 9 | S1 left | Most lateral point of the sacral canal |
| 10 | S1 left | Most inferomedial point of the superior articular facet |
| 11 | dorsal right | S1 foramen sacrale, superior most point |
| 12 | dorsal right | S2 foramen sacrale, medialmost point |
| 13 | dorsal right | S3 foramen sacrale, medialmost point |
| 14 | dorsal right | S4 foramen sacrale, medialmost point |
| 15 | dorsal left | S1 foramen sacrale, superior most point |
| 16 | dorsal left | S2 foramen sacrale, medialmost point |
| 17 | dorsal left | S3 foramen sacrale, medialmost point |
| 18 | dorsal left | S4 foramen sacrale, medialmost point |
| 19 | ventral | S1 midpoint of first transverse line |
| 20 | ventral | S2 midpoint of second transverse line |
| 21 | ventral | S3 midpoint of third transverse line |
| 22 | ventral | S4 midpoint of fourth transverse line |
| 23 | ventral | S5 midpoint of fifth transverse line |
| 24 | ventral right | S1 foramen sacrale, medialmost point at first transverse line |
| 25 | ventral right | S2 foramen sacrale, medialmost point at second transverse line |
| 26 | ventral right | S3 foramen sacrale, medialmost point at third transverse line |
| 27 | ventral right | S4 foramen sacrale, medialmost point at fourth transverse line |
| 28 | ventral right | Lateralmost point of the apex of the sacrum |
| 29 | ventral left | S1 foramen sacrale, medialmost point at first transverse line |
| 30 | ventral left | S2 foramen sacrale, medialmost point at second transverse line |
| 31 | ventral left | S3 foramen sacrale, medialmost point at third transverse line |
| 32 | ventral left | S4 foramen sacrale, medialmost point at fourth transverse line |
| 33 | ventral left | Lateralmost point of the apex of the sacrum |
| 34 | Auricular facet right | Auricular surface, most ventral point |
| 35 | Auricular facet right | Auricular surface, most dorsal point |
| 36 | Auricular facet right | Auricular surface, deepest point of dorsal notch |
| 37 | Auricular facet right | Auricular surface, most caudal point |
| 38 | ventral right | Superior end of lateral crest |
| 39 | Auricular facet left | Auricular surface, most ventral point |
| 40 | Auricular facet left | Auricular surface, most dorsal point |
| 41 | Auricular facet left | Auricular surface, deepest point of dorsal notch |
| 42 | Auricular facet left | Auricular surface, most caudal point |
| 43 | ventral left | Superior end of lateral crest |
| Curve | Location | Description |
| 1 | Base | Superior articular surface curve |
| 2 | S1 right | Right sacral ala curve |
| 3 | Auricular surface right | Right auricular surface |
| 4 | Right | Right lateral edge |
| 5 | Base | Left sacral ala curve |
| 6 | Auricular surface left | Left auricular surface |
| 7 | Left | Left lateral edge |
| Abbreviation: S, sacral vertebrae | | |

| **Table S3 –** Percentage of explained variance for the first three PCs after geometric morphometric analysis of the landmark subsets for the first sacral vertebra (S1), the first and second sacral vertebra (S2) and auricular surface, in shape space | | | |
| --- | --- | --- | --- |
| Analysis in shape space | PC1 | PC2 | PC3 |
| S1 | 30.2 | 14.6 | 9.5 |
| S2 | 24.1 | 13.2 | 10 |
| Auricular surface | 21.0 | 14.6 | 11.6 |
| Abbreviation: PC, principal component | | | |

| \| **Table S4–** Shape variation contributions of geography, sex and size using an ANCOVA analysis, including the significance of these variables considered as two-way and three-way interaction terms for the entire sample and the European and African subsamples. \| \| \| \| \| \| \| \| \| --- \| --- \| --- \| --- \| --- \| --- \| --- \| --- \| \|  \| df \| SS \| MS \| R^2^ \| F \| Z \| Pr(>F) \| \| **Complete Sample** \|  \|  \|  \|  \|  \|  \|  \| \| lnCS \| 1 \| 0.01413 \| 0.014130 \| 0.01572 \| 1.6828 \| 1.5037 \| 0.073 \| \| Sex \| 1 \| 0.04239 \| 0.042390 \| 0.04715 \| 5.0485 \| 3.5424 \| 0.001 ** \| \| Geo \| 3 \| 0.04651 \| 0.015505 \| 0.05174 \| 1.8466 \| 2.4411 \| 0.009 ** \| \| lnCS:Sex \| 1 \| 0.00973 \| 0.009729 \| 0.01082 \| 1.1587 \| 0.5349 \| 0.296 \| \| lnCS:Geo \| 3 \| 0.02593 \| 0.008642 \| 0.02884 \| 1.0292 \| 0.2763 \| 0.407 \| \| Sex:Geo \| 3 \| 0.03327 \| 0.011089 \| 0.03701 \| 1.3207 \| 1.1580 \| 0.121 \| \| lnCS:Sex:Geo \| 3 \| 0.01330 \| 0.004434 \| 0.01480 \| 0.5280 \| -2.4343 \| 0.990 \| \| Residuals \| 85 \| 0.71370 \| 0.008397 \| 0.79392 \|  \|  \|  \| \| Total \| 100 \| 0.89896 \|  \|  \|  \|  \|  \| \| **European Sample** \|  \|  \|  \|  \|  \|  \|  \| \| LnCS \| 1 \| 0.01952 \| 0.019517 \| 0.03409 \| 2.3852 \| 2.038 \| 0.021* \| \| lnCS:Sex \| 1 \| 0.03751 \| 0.037510 \| 0.06552 \| 4.5842 \| 3.063 \| 0.001 ** \| \| Residuals \| 63 \| 0.51549 \| 0.008182 \| 0.90039 \|  \|  \|  \| \| Total \| 65 \| 0.57252 \|  \|  \|  \|  \|  \| \| **African Sample** \|  \|  \|  \|  \|  \|  \|  \| \| lnCS \| 1 \| 0.005811 \| 0.0058112 \| 0.03240 \| 0.7215 \| -0.58906 \| 0.709 \| \| lnCS:Sex \| 1 \| 0.020517 \| 0.0205170 \| 0.11439 \| 2.5473 \| 2.13248 \| 0.021 * \| \| Residuals \| 19 \| 0.153034 \| 0.0080544 \| 0.85321 \|  \|  \|  \| \| Total \| 21 \| 0.179362 \|  \|  \|  \|  \|  \| |
| --- | --- | --- | --- | --- | --- | --- | --- | --- | --- | --- | --- | --- | --- | --- | --- | --- | --- | --- | --- | --- | --- | --- | --- | --- | --- | --- | --- | --- | --- | --- | --- | --- | --- | --- | --- | --- | --- | --- | --- | --- | --- | --- | --- | --- | --- | --- | --- | --- | --- | --- | --- | --- | --- | --- | --- | --- | --- | --- | --- | --- | --- | --- | --- | --- | --- | --- | --- | --- | --- | --- | --- | --- | --- | --- | --- | --- | --- | --- | --- | --- | --- | --- | --- | --- | --- | --- | --- | --- | --- | --- | --- | --- | --- | --- | --- | --- | --- | --- | --- | --- | --- | --- | --- | --- | --- | --- | --- | --- | --- | --- | --- | --- | --- | --- | --- | --- | --- | --- | --- | --- | --- | --- | --- | --- | --- | --- | --- | --- | --- | --- | --- | --- | --- | --- | --- | --- | --- | --- | --- | --- | --- | --- | --- | --- | --- | --- | --- | --- | --- | --- | --- | --- | --- | --- | --- | --- | --- | --- | --- | --- | --- | --- | --- | --- | --- | --- | --- | --- | --- | --- | --- | --- | --- | --- | --- | --- |

* p of 0.05 or less; ** p of 0.01 or less

Abbreviations: df, degree of freedom; SS, sum-of-squared Procrustes distances; MS, mean squares (sum-of-squares divided by the corresponding df); F, F ratio; Z, z score; Pr(>F), p value; lnCS, natural logarithm of centroid size; Geo, geographical origin
