# Supplementary figures and images for: Sex classification using the human sacrum: Geometric morphometrics versus conventional approaches
Source: PLoS One. 2022 Apr 6;17(4):e0264770. doi: 10.1371/journal.pone.0264770 (PMC8986015; doi:10.1371/journal.pone.0264770)

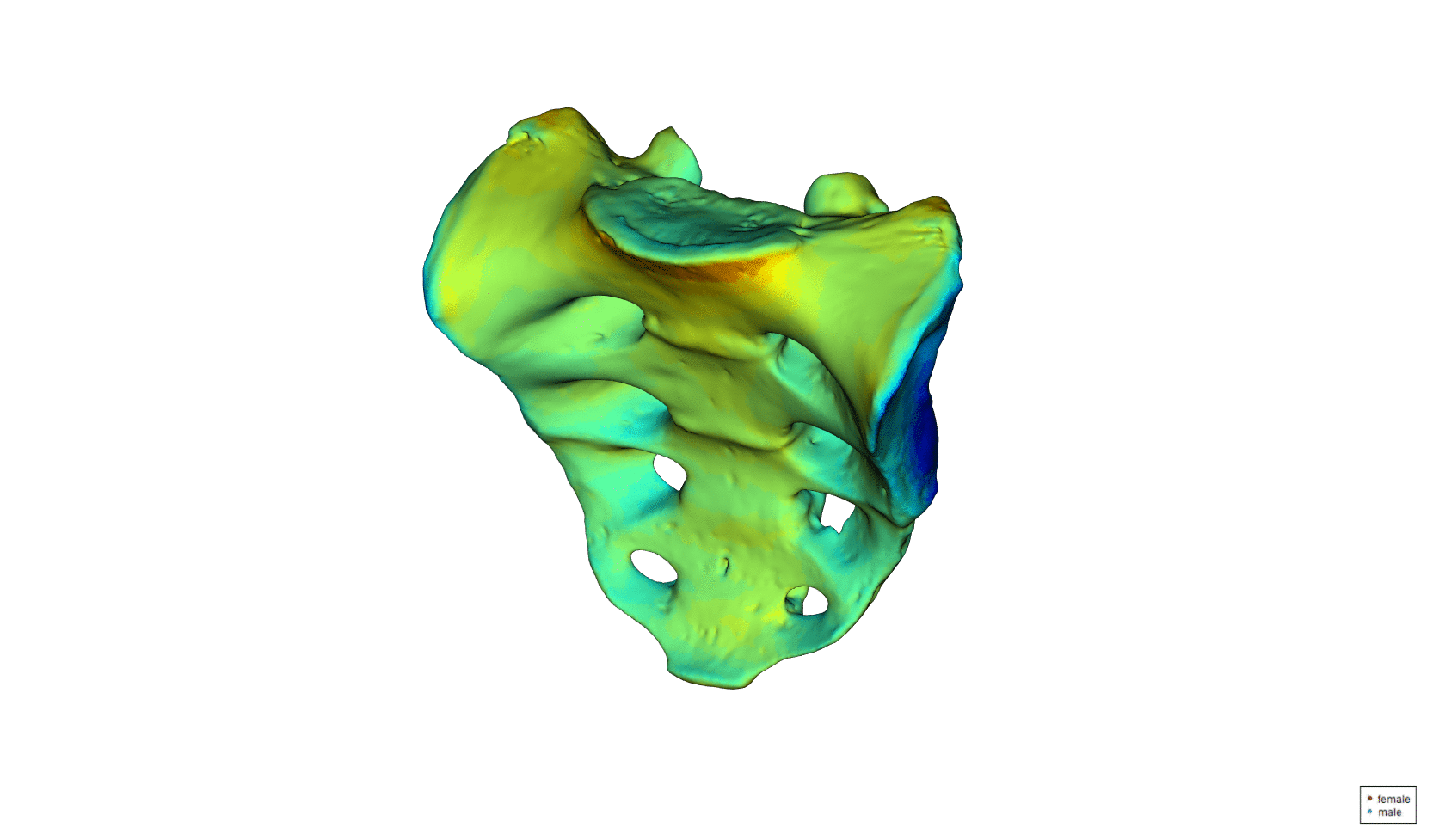

Supplement: S1 Video — (GIF) [file pone.0264770.s002.gif]

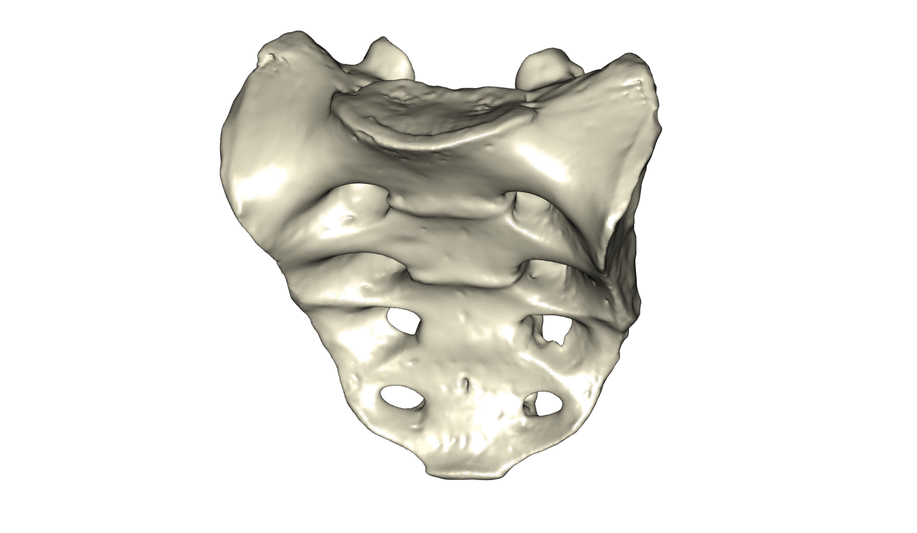

Supplement: S2 Video — (GIF) [file pone.0264770.s003.gif]

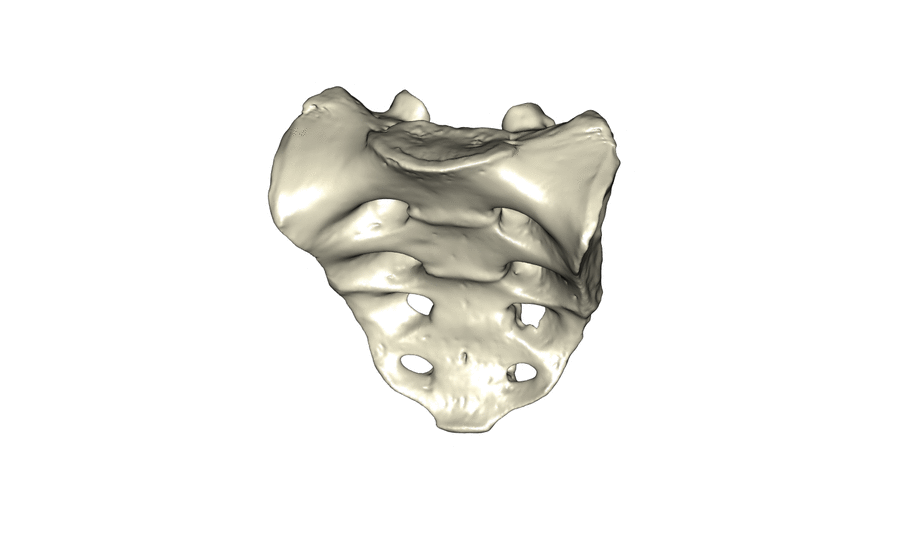

Supplement: S3 Video — (GIF) [file pone.0264770.s004.gif]
